# Humoral Immune Variability in Twin Sisters With SKIV2L-Associated Trichohepatoenteric Syndrome

**DOI:** 10.7759/cureus.115524

**Published:** 2026-08-31

**Authors:** Shreena Reddy, Curtis S Pacheco, Vishaka R Hatcher

**Affiliations:** 1 Pediatrics, Joint Base Charleston, Charleston, USA; 2 Allergy and Immunology, Wilford Hall Ambulatory Surgical Center, San Antonio, USA; 3 Allergy and Immunology, Eglin Air Force Base, Destin, USA

**Keywords:** humoral immunity, immunodeficiency, memory b cells, skiv2l, specific antibody deficiency, trichohepatoenteric syndrome, vaccine response

## Abstract

Trichohepatoenteric syndrome (THES) is a rare autosomal recessive disorder caused by pathogenic variants in tetratricopeptide repeat domain 37 (TTC37), designated THES-I, or Ski2-like RNA helicase (SKIV2L), designated THES-II, and characterized by infantile diarrhea, growth impairment, developmental delay, liver disease, hair abnormalities, and variable immune dysfunction. Although immunologic abnormalities have increasingly been recognized, the functional humoral phenotype of SKIV2L-associated THES remains incompletely characterized.

We report twin sisters with compound heterozygous SKIV2L variants, consistent with THES-II, who underwent comprehensive immunologic evaluation after recurrent infections and concern for immune dysfunction. Both twins demonstrated persistently elevated serum immunoglobulin A concentrations, reduced memory B-cell populations, decreased plasmablasts, and predominantly nonprotective baseline pneumococcal antibody titers despite routine childhood immunization. However, selected quantitative and antigen-specific humoral findings differed between the twins.

Twin 1 had infantile hypogammaglobulinemia that normalized by approximately three years of age without immunoglobulin replacement therapy but later demonstrated absent tetanus and diphtheria antibodies and a severely impaired response to pneumococcal polysaccharide vaccination, with protection against only 1 of 23 serotypes. Twin 2 never developed hypogammaglobulinemia and retained a protective diphtheria antibody response. However, tetanus antibody was absent and formal assessment of her polysaccharide vaccine responsiveness was limited by unavailable post-vaccination titers.

These cases demonstrate intrafamilial variability in selected humoral immune features despite a shared disease-causing genotype and show that clinically important antibody dysfunction may not be apparent from quantitative immunoglobulin measurements alone. Comprehensive evaluation of vaccine responses and memory B-cell maturation may improve recognition and individualized management of immune dysfunction in patients with THES-II.

## Introduction

Trichohepatoenteric syndrome (THES), also known as syndromic diarrhea, is an exceedingly rare autosomal recessive disorder with an estimated prevalence of approximately one per 1,000,000 live births [1]. The syndrome is classically characterized by severe infantile diarrhea, failure to thrive, developmental delay, distinctive hair abnormalities, liver disease, and variable immunologic dysfunction [1]. THES results from pathogenic variants in tetratricopeptide repeat domain 37 (TTC37), designated THES-I, or Ski2-like RNA helicase (SKIV2L), designated THES-II. Both genes encode components of the superkiller (SKI) complex, which functions in cytoplasmic RNA surveillance and exosome-mediated RNA degradation [2]. Experimental studies further demonstrate that SKIV2L is required for early B-cell development and immunoglobulin gene expression, providing a biologic link between SKI complex dysfunction and impaired antibody production [3].

Reported immunologic abnormalities in THES include hypogammaglobulinemia, impaired vaccine responses, reduced B-cell numbers or memory B-cell populations, and variable T-cell and NK-cell abnormalities [4-6]. However, the relationship between quantitative immunoglobulin levels, B-cell maturation, and functional antibody responses remains poorly characterized, particularly in SKIV2L-associated THES-II, for which the available literature consists largely of individual cases and small cohorts. T-cell-dependent protein-antigen responses, such as tetanus and diphtheria antibodies, and responses to T-cell-independent pneumococcal polysaccharide antigens provide complementary measures of humoral immune function and may identify functional antibody impairment despite preserved quantitative immunoglobulin levels [7].

We describe twin sisters with compound heterozygous SKIV2L variants consistent with THES-II who shared multiple immunophenotypic abnormalities but demonstrated differences in quantitative IgG trajectories and selected antigen-specific antibody responses. Their comparison provides an opportunity to examine intrafamilial variability in a rare disease and highlights the clinical value of comprehensive immune assessment. To our knowledge, these are the first reported cases of SKIV2L-associated THES-II in the United States.

## Case presentation

Twin 1 is a 4-year-old girl born at 35 weeks’ gestation as part of a twin pregnancy. Her early clinical course was notable for failure to thrive and significant feeding difficulties, with subsequent development of severe dysphagia requiring gastrostomy tube placement in January 2024. She remains gastrostomy dependent with ongoing gastroenterology and nutrition follow-up. She also developed atopic dermatitis and global developmental delay affecting speech and language, gross and fine motor development, and adaptive functioning.

Her infectious history included hospitalization for respiratory syncytial virus infection during infancy, followed by pneumonia and two episodes of acute otitis media. Chromosomal sequencing analysis (20,096-gene panel; gene sequencing with deletion and duplication analysis and genome-wide copy-number analysis) identified compound heterozygous SKIV2L variants, NM_006929.5:c.1120C>T (p.Arg374*), classified as pathogenic, and NM_006929.5:c.1079C>T (p․Ser360Leu), classified as likely pathogenic (Fulgent Genetics, El Monte, CA). Maternal testing confirmed inheritance of the c.1120C>T (p.Arg374*) variant; the c.1079C>T (p․Ser360Leu) variant was not maternally inherited, and segregation analysis supported an in-trans configuration. Paternal testing was not documented. The patient additionally carried a maternally inherited likely pathogenic FLG variant, NM_002016.2:c.11452C>T (p.Gln3818*), and a heterozygous NOD2 increased-risk allele, c.2104C>T (p.Arg702Trp). A three-generation family history was otherwise noncontributory, with no reported miscarriages, previous affected pregnancies, or relatives with similar clinical features.

Preliminary immunologic evaluation during infancy demonstrated marked hypogammaglobulinemia, with serum IgG concentrations of 146 mg/dL and 159 mg/dL. By approximately 3 years of age, serum IgG had spontaneously normalized to 900 mg/dL (reference range, 441-1,135 mg/dL) and remained within the age-adjusted laboratory reference range on subsequent testing at 577 mg/dL (reference range, 453-916 mg/dL). Despite normalization of quantitative IgG levels without immunoglobulin replacement therapy, her infectious history prompted further immunologic evaluation.

Baseline pneumococcal serotype-specific antibody titers were predominantly nonprotective. Following PPSV23 administration, post-vaccination testing demonstrated a severely impaired response, with protective antibody levels against only 1 of 23 serotypes. Tetanus and diphtheria antitoxoid IgG concentrations were both <0.10 IU/mL despite prior routine immunization. Flow cytometry demonstrated reduced memory and class-switched memory B-cell populations, with additional abnormalities involving plasmablasts and transitional B cells. Serum IgA was persistently elevated, reaching 756 mg/dL.

Twin 2, the 4-year-old twin sister of Twin 1, carried the same compound heterozygous SKIV2L variants and also had developmental delay and atopic dermatitis. Unlike her sister, she did not require gastrostomy tube placement and was subsequently diagnosed with autism spectrum disorder. Her infectious history included pneumonia, bacteremia, and recurrent acute otitis media requiring bilateral tympanostomy tube placement in 2023 and repeat placement in 2026.

Neither twin developed intractable diarrhea or clinically significant hepatic disease classically associated with THES-II [1]. Sparse, thin hair and mottled skin were noted on genetic evaluation; characteristic hair-shaft abnormalities and significant alopecia were not noted.

Unlike Twin 1, Twin 2 never demonstrated hypogammaglobulinemia. Serum IgG remained preserved, including values of 1,065 mg/dL and 1,134 mg/dL on follow-up testing. Serum IgA was persistently elevated, reaching 795 mg/dL. Flow cytometry demonstrated decreased total B cells, reduced memory and class-switched memory B-cell populations, decreased plasmablasts, and increased NK-cell numbers.

Functional humoral assessment showed predominantly nonprotective baseline pneumococcal serotype-specific antibody titers. Tetanus antitoxoid IgG was <0.10 IU/mL, whereas diphtheria antitoxoid IgG was protective at 1.64 IU/mL. PPSV23 was administered; however, post-vaccination pneumococcal titers were not obtained because of a laboratory collection error, precluding formal assessment of polysaccharide vaccine responsiveness. Repeat functional antibody assessment was therefore being considered. The clinical, genetic, and immunologic findings are summarized in Table 1.

**Table 1:** Comparative immunologic findings in twin sisters with SKIV2L-associated THES-II THES, trichohepatoenteric syndrome; FLG, filaggrin; IgA, immunoglobulin A; IgG, immunoglobulin G; IgRT, immunoglobulin replacement therapy; NOD2, nucleotide-binding oligomerization domain-containing protein 2; PPSV23, 23-valent pneumococcal polysaccharide vaccine

Characteristic	Twin 1	Twin 2
Molecular findings	Compound heterozygous SKIV2L variants: c.1120C>T (p.Arg374*), pathogenic; c.1079C>T (p․Ser360Leu), likely pathogenic; FLG c.11452C>T (p.Gln3818*), likely pathogenic; NOD2 c.2104C>T (p.Arg702Trp), increased-risk allele	Same compound heterozygous SKIV2L variants
Shared immunologic findings		
Serum IgA	Persistently elevated; peak 756 mg/dL (lab ref 22-159)	Persistently elevated; peak 795 mg/dL (lab ref 20-100)
Memory B-cell abnormalities	CD27+ memory B cells 3.0% (ref 4.6-49.1%); class-switched memory B cells 0.6% (ref 1.9-30.4%)	CD27+ memory B cells 4.0% (ref 4.6-49.1%); class-switched memory B cells 1.7% (ref 1.9-30.4%); plasmablasts 0.6% (ref 2.9-51.8%)
Baseline pneumococcal antibody status	Predominantly nonprotective; most serotype-specific titers <0.1-0.3 µg/mL	Predominantly nonprotective; most serotype-specific titers <0.1-0.3 µg/mL
Comparative humoral findings		
Quantitative IgG / IgG subclasses	146-159 mg/dL in infancy; later 900 mg/dL (ref 441-1,135) and 577 mg/dL (ref 453-916), normalized without IgRT; IgG subclass data not available	No hypogammaglobulinemia; 1,065 mg/dL (ref 441-1,135) and 1,134 mg/dL (ref 453-916); IgG subclass data not available
Protein vaccine responses	Tetanus <0.10 IU/mL; diphtheria <0.10 IU/mL	Tetanus <0.10 IU/mL; diphtheria 1.64 IU/mL
Polysaccharide vaccine responses	Severely impaired post-PPSV23 response: 1/23 protective serotypes ≥ 1.3 µg/mL(19F 2.0 µg/mL)	PPSV23 challenge completed; post-vaccination titers unavailable because of laboratory collection error
Overall humoral interpretation	Severe functional antibody impairment despite normalized IgG; findings extend beyond isolated polysaccharide deficiency	Selected antigen-specific antibody abnormalities; polysaccharide responsiveness incompletely assessed
Management considerations	IgRT and prophylactic antibiotics considered; currently deferred	Repeat functional antibody assessment under consideration

## Discussion

Reported immunologic abnormalities in THES are heterogeneous and include hypogammaglobulinemia, impaired vaccine responses, reduced switched memory B-cell populations, elevated IgA levels, and abnormalities of T-cell and NK-cell function [4-6]. The reported frequency of immune dysregulation has varied considerably, from 5 of 24 patients (21%) in one multicenter cohort to 39 of 44 patients (88.6%) in an earlier review [1,6], further illustrating the heterogeneity of immune involvement in THES.

Our cases build upon these observations and further characterize the immunologic spectrum of SKIV2L-associated THES-II. Both twins demonstrated persistent hyper-IgA and abnormalities of memory B-cell maturation, including reduced memory and class-switched memory B-cell populations. These shared findings, together with prior reports of B-cell dysfunction in THES [3-6], may represent an emerging pattern of humoral immune dysregulation; however, additional cases are needed to determine whether these findings constitute a recurring THES-II immunophenotype. In these twins, hyper-IgA coexisted with abnormalities of B-cell maturation and antigen-specific antibody responses, indicating that elevated IgA should not be interpreted as evidence of preserved humoral function.

Despite these shared immunophenotypic features, the twins demonstrated partially discordant humoral immune profiles. Twin 1 had marked hypogammaglobulinemia during infancy, with IgG concentrations of 146 and 159 mg/dL, followed by spontaneous normalization without immunoglobulin replacement therapy. Despite subsequent normalization of IgG, she had absent tetanus and diphtheria antibodies and a severely impaired response to PPSV23, with protective titers to only 1 of 23 serotypes post-vaccination. These findings demonstrate substantial impairment of antigen-specific antibody production despite normalization of quantitative IgG. Although the severe polysaccharide response is compatible with a specific antibody deficiency phenotype, the concurrent absence of protein-antigen responses and prior hypogammaglobulinemia suggest broader humoral immune dysfunction rather than an isolated defect in pneumococcal polysaccharide responsiveness [7].

Twin 2, in contrast, maintained preserved quantitative IgG and retained a protective diphtheria antibody response, although tetanus antibody was absent in both sisters and baseline pneumococcal titers were predominantly nonprotective. Formal assessment of Twin 2's polysaccharide responsiveness could not be completed because post-PPSV23 titers were unavailable. Thus, the clearest differences between the twins were their quantitative IgG trajectories and diphtheria-specific antibody responses, while direct comparison of polysaccharide vaccine responsiveness was not possible.

The coexistence of normalized serum IgG with markedly impaired vaccine-specific antibody responses in Twin 1 illustrates that quantitative immunoglobulin concentrations alone may not fully reflect functional humoral competence [7]. Assessment of antigen-specific antibody responses and B-cell maturation may therefore provide clinically useful information beyond routine immunoglobulin measurement in patients with THES-II.

Twin 1 additionally carried a likely pathogenic FLG variant and a NOD2 increased-risk allele. Both genes have roles in epithelial barrier integrity and mucosal immune regulation [8,9]. Although causality cannot be inferred from these variants, additional genetic modifiers could potentially contribute to phenotypic differences between the twins. This possibility remains speculative and requires further study.

Overall, these cases support individualized immunologic evaluation in THES-II. Similar abnormalities of IgA and B-cell maturation did not correspond to identical functional antibody findings in these twins. Comprehensive assessment incorporating quantitative immunoglobulins, vaccine-specific antibody responses, and B-cell phenotyping may better characterize clinically meaningful immune dysfunction in this rare disorder.

## Conclusions

These cases demonstrate intrafamilial variability in selected humoral immune features among twin sisters with SKIV2L-associated THES-II. Both shared marked hyper-IgA and memory B-cell abnormalities, while quantitative IgG trajectories and selected antigen-specific antibody responses differed. Because post-PPSV23 titers were unavailable for Twin 2, polysaccharide vaccine responsiveness could not be directly compared. These shared abnormalities may represent an emerging pattern that warrants further study. Comprehensive assessment of quantitative immunoglobulins, vaccine-specific antibody responses, and B-cell maturation may help guide individualized immune evaluation.
